# The financial burden of diabetes-related foot disease in Australia: a systematic review

**DOI:** 10.1186/s13047-023-00688-y

**Published:** 2023-12-27

**Authors:** Nicoletta Frescos, Lucy Stopher, Shirley Jansen, Michelle R. Kaminski

**Affiliations:** 1 Austin Health, Melbourne, VIC Australia; 2 Discipline of Podiatry, School of Allied Health, Human Services and Sport, La Trobe University, Melbourne, VIC Australia; 3 Sir Charles Gairdner Hospital, Perth, WA Australia; 4 Curtin Medical School, Curtin University, Perth, WA Australia; 5 Harry Perkins Institute of Medical Research, Perth, WA Australia; 6 Department of Podiatry, Monash Health, Melbourne, VIC Australia; 7 School of Primary and Allied healthcare, Monash University, Melbourne, VIC Australia

**Keywords:** Amputation, Cost analysis, Diabetic foot, Diabetes-related foot disease, Foot ulcer

## Abstract

**Background:**

Diabetes-related foot disease (DFD) is a common, costly, and severe complication of diabetes mellitus. DFD is associated with high rates of morbidity and mortality and poses a significant burden on patients, healthcare systems and society. While the detrimental impact of DFD is widely recognised, the precise financial implications of its management in Australia remain unclear due to inconsistent and inconclusive contemporary data. Therefore, the aim of this review was to identify, summarise and synthesise existing evidence to estimate the costs associated with DFD management in Australia.

**Methods:**

Searches were conducted in MEDLINE, Embase, AMED, CINAHL, Joanna Briggs Institute EBP, and the Cochrane Library from November 2011 to July 2023. Australian studies investigating costs associated with DFD management were eligible for inclusion. Two independent reviewers performed the study selection, data extraction and quality assessment steps. The Consolidated Health Economic Evaluation Reporting Standards (CHEERS 2022) checklist was used to assess study quality. A descriptive analysis was performed due to limited existing evidence and large heterogeneity between study populations to conduct meta-analyses.

**Results:**

Three economic evaluations were included in the review. One study was rated as ‘poor’, one as ‘very good’ and one as ‘excellent’ when assessed against the CHEERS checklist. The estimated cost of DFD management varied between studies and comparisons were not possible due to the different methodological approaches and data sources. The studies were unable to provide an overall cost of DFD with respect to all aspects of care as they did not capture the multi-faceted level of care throughout the entire patient journey between sectors and over time.

**Conclusion:**

There is limited contemporary evidence for the costs associated with DFD management within Australia, particularly related to direct costs and resource utilisation. Further research into the economic impact of DFD management is needed to inform optimisation of national service delivery and improve health outcomes for individuals with DFD in Australia. Integrating real-world data on impact of clinical interventions with parallel economic evaluation could be a valuable approach for future research, which would offer a more comprehensive understanding of the clinical and economic outcomes beyond solely model-based evaluations.

**Trial registration:**

PROSPERO Registration No. CRD42022290910.

## Introduction

Diabetes mellitus affects over 537 million people worldwide and is associated with high morbidity and mortality [1]. A debilitating sequela of this chronic condition is diabetes-related foot disease (DFD), which is a limb-threatening complication encompassing diabetes-related foot ulceration (DFU), infection, ischaemia, and lower limb amputation [2]. DFD is a leading cause of hospitalisation, lower limb amputations, and is a large contributor to the global disability burden [2–9].

Prevalence studies have shown that DFU affects 6.3% of the global diabetes population [8] and has a lifetime incidence in people with diabetes between 19 to 34% [2, 10]. DFU recurrence is also frequent, where 40% of ulcers will reoccur within one year and 65% within three years [2, 10]. In Australia, the prevalence of DFD ranges from 1.2 to 1.5%, while the incidence of diabetes-related lower limb amputations is between 5.2 to 7.2 per 1000 person-years. DFD-related hospital admissions are common in Australians with diabetes and range from 5.2 to 36.6 per 1000 person-years [11].

The financial burden associated with DFD management varies greatly between countries and is contingent on many variables such as patient factors (e.g. ulcer severity), interventions used, and the length of stay for DFD-related hospital admissions for specialised care and rehabilitation [11]. Healthcare costs associated with DFD management may include medical appointments, wound care products and consumables, medications, pressure offloading and prosthetic devices, diagnostic tests, hospitalisations, and surgical interventions [12, 13].

In the United States (US), the direct annual costs for diabetes management is estimated at USD$237 billion, where one third is attributable to DFD [14]. Expenditure for DFD in the United Kingdom (UK) is estimated to be between £837 to £962 million per year (data from 2014–2015) [13]. In Europe, the total direct and indirect costs associated with DFD management (at the individual level) is between €7,722 to €20,064 per annum [15]. While the economic impact of DFD management has been well established in other countries [13–15], contemporary cost data within Australia remains ambiguous. Foot disease is considered to be one of the most costly acute complications of diabetes [16, 17]. Estimates of the direct costs for DFD management to the public hospital system and overall health system in Australia have been reported to be AUD$348 million and AUD$1.57 billion, respectively [18]. However, this data is based on modelling from a point prevalence study of less than 900 inpatients and extrapolated nation-wide. This included assumptions that every hospital had 600 inpatient beds and the average stay for DFD was 29 occupied bed days across all sites. As these assumptions resulted in a total of 4,385 hospital total bed days, this ultimately led to the AUD$1.57 billion cost estimate [19].

Considering the increasing prevalence of DFD and high rates of recurrence, it is unsurprising that the costs associated with its management are substantial. Hence, it is imperative to ascertain the *current* financial burden of DFD management within Australia. This is particularly important for ensuring optimal national service delivery and policy development for the provision of prevention strategies and best practice management. Consequently, the aim of this systematic review was to identify, summarise and synthesise existing evidence to estimate the costs associated with DFD management in Australia.

## Methods

### Registration

This systematic review was prospectively registered with The International Prospective Register of Systematic Reviews (PROSPERO)—Registration No. CRD42022290910. Detailed methods have been published elsewhere [20]. This review is reported in accordance with the Preferred Reporting Items for Systematic Review and Meta-analysis (PRISMA) guidelines [21].

### Search strategy

Searches were conducted in MEDLINE (Ovid), Embase (Ovid), AMED (Ovid), CINAHL (EBSCO), Joanna Briggs Institute EBP (Ovid), and the Cochrane Library from 1 November 2011 to 23 November 2021 without language restriction. The MEDLINE search strategy is available in the protocol article [20]. To ensure literature saturation, citation tracking was performed using Google Scholar and reference lists were screened for studies not identified in the initial search. The searches were re-run to 20 July 2023 (i.e. search timeframe 1 November 2011 to 20 July 2023) to ensure any new studies were captured in this review prior to publication.

### Eligibility criteria

Peer-reviewed Australian studies investigating costs associated with DFD management between November 2011 to November 2021 were eligible for inclusion. Searches were re-run to 20 July 2023 to ensure all contemporary data were included. A ten-year timeframe was chosen to ensure *current* cost estimates within Australia were captured. For clarity, this timeframe was extended by 20 months (i.e. searches were re-run up to July 2023) prior to manuscript submission for publication.

The population of interest were adults with DFD (i.e. DFU, infection, ischaemia, amputation) in any clinical setting. All reported costs for DFD management were considered, however, costs of particular interest were visits to a healthcare professional, consumables (e.g., wound dressings, footwear, offloading and prosthetic devices), anti-infective agents, diagnostic tests/imaging, and/or surgical procedures (e.g., debridement, amputation). Single case reports/studies/series, expert opinion level V studies, protocols, abstracts without full text, conference proceedings, literature reviews, case–control, validity or reliability studies, letters, editorials, notes, and short surveys were excluded.

### Data management

All citations were initially exported into EndNote 20 (Thomson Reuters, New York, USA) for automated removal of duplicates. To conduct the study selection process, the remaining unique citations were imported into the Covidence systematic review software (Veritas Health Innovation, Melbourne, Australia) and any further identified duplicates were removed.

### Study selection

The Covidence systematic review software (Veritas Health Innovation, Melbourne, Australia) was used by two independent reviewers during the study selection process to screen titles and abstracts (NF and LS) and to perform the full-text review (NF and MRK). Conflicts were discussed and resolved at each stage of the study selection process. The above method was repeated for the citation tracking and bibliographic reference scanning steps [20].

### Data extraction

A pre-specified data extraction form was implemented to extract relevant study information, participant characteristics and reported costs associated with DFD management (Table 1). Data extraction was performed by two authors (NF and MRK) and checked for accuracy and omissions by another author (LS). For further information on the data extraction process, we refer the reader to our protocol article [20]. On our request, authors from one study [22] provided their raw cost data.

**Table 1:** Characteristics of included studies

**Study information**																	
**Author (Year)**	**Study design**	**Sample Size**			**Study population / setting**			**Inclusion / exclusion**			**Aims / objectives**			**Intervention / comparator**	**Study outcomes**	**Model description**	**Statistical analysis**
Cheng et al. (2017) [22]	Cost-effectiveness analysis	Hypothetical cohort of people with DM at high risk of DFU Cohort based on patients registered with the National Diabetes Services Scheme (2015) in Australia			Simulated cohort of people with DM at high risk of developing DFUs in Australia Simulations were separated for differing age groups. The distribution of diabetes among the age groups was informed by the Australian Health Survey 2011–2014			People with DM at high risk of DFU (i.e. those with previous DFU or amputation)			To examine the costs and health outcomes associated with implementing optimal guideline-based care compared with usual care in people at high risk of DFUs in Australia			**Optimal care**: Components of foot examination, debridement, wound dressings, pressure offloading, infection management and multidisciplinary care **Usual care:** A mix of largely uncoordinated set of services in the community	Expected costs, cost-effectiveness and QALYs associated with optimal care versus usual care	Markov model: - 5-years - 1-month cycles - 60 cycles in total - 7 possible health states	- Markov model - Scenario analysis - Probabilistic sensitivity analysis
Graves and Zheng (2014) [25]	Economic evaluation	12,839 (SD, 3,534) cases of DFU in all hospitals in Australia 516 (SD, 141) cases of DFU in residential care setting in Australia			Cohort of patients with pressure ulcer, DFU, venous ulcer or artery insufficiency ulcer located in hospital and residential care settings in Australia for 2010–2011			People with either a pressure ulcer, DFU, venous ulcer or artery insufficiency ulcer			To estimate the direct healthcare costs of chronic wounds in hospital and residential care settings in Australia			Not applicable	Direct healthcare costs of chronic wounds in hospital and residential care settings	Probabilistic model to estimate direct healthcare costs	- Probabilistic analysis
Zhang et al. (2023) [26]	Cost-effectiveness analysis	Overall cohort of patients with DFU (n = 3,385) who presented to Diabetic Foot Services in Queensland, Australia followed up for at least 3 years Model included 3,122 patients with care data to derive the events and corresponding time-to-event parameters			A prospective cohort of patients with DFU attending multi-site outpatient Diabetic Foot Services in Queensland, Australia between 1/7/2011 to 1/6/2016			People with DFU attending Diabetic Foot Services			Primary aim: To estimate the costs and QALYs associated with complete adherence to guideline-based care, compared with current practice Secondary aim: To estimate the costs, cost-effectiveness and QALYs associated with increasing levels of guideline-based care, compared with current practice			**Guideline-based care**: Components of foot examination, debridement, wound dressings, offloading, infection management and multidisciplinary care **Current practice (sub-optimal care):** 30% of patients receiving guideline-based care and 70% receiving sub-optimal care (i.e. all other care that does meet the definition of guideline-based care)	Expected costs, cost-effectiveness and QALYs associated with guideline-based care versus current practice (i.e. sub-optimal care)	Discrete event simulation model - 3-years - 6 possible health states	- Discrete event simulation model - Parametric survival analysis - Probabilistic sensitivity analysis
**Participant characteristics**																	
**Author (Year)**	**Age**		**Sex**			**Diabetes type/duration**				**Comorbidities**		**Clinical state of DFD**				**Ulcer characteristics**	
Cheng et al. (2017) [22]	Age groups (years): - 35–54 - 55–74 - 75 +		Not reported			Not reported				Not reported		Markov model health states included: no DFU, uncomplicated DFU, complicated DFU with infection, post minor amputation, infected post minor amputation, post major amputation, and death				As per the health states used in the Markov model	
Graves and Zheng (2014) [25]	≥ 15 years for hospital separations ≥ 65 years for aged care residents		Not reported			Not reported				Not reported		Not reported				Not reported	
Zhang et al. (2023) [26]	62 (SD, 13) years		Male: 2,350 (69.4%) Female: 1,035 (30.6%)			Type 1 DM: 314 (9.3%) Type 2 DM: 3,071 (90.7%) Diabetes duration: 16.4 (SD, 10.7) years HbA1c: 8.52 (SD, 2.44)				DM, hypertension, dyslipidaemia, CVD, CKD, ESRD, smoking		Markov model discrete episodes of disease included: healed DFU, recurrent DFU, hospitalisation (no amputation), minor amputation, major amputation, and death				Ulcer size: < 1cm^2^ = 1,559 (46.1%) 1-3cm^2^ = 643 (19.0%) > 3cm^2^ = 551 (16.3%) Deep ulcer: 518 (15.3%) Infection: Nil = 2,226 (65.8%) Mild = 753 (22.2%) Moderate to systemic = 405 (12.0%)	
**Cost of DFD**																	
**Author (Year)**	**Type and frequency of treatments**								**Provision of treatment**				**Unit costs of treatment / model inputs**			**Data sources**	
Cheng et al. (2017) [22]	**Usual care:** If ‘*uncomplicated DFU*’, patients assumed to receive: - One-off initial assessment by GP for risk of amputation - Medical checks by GP twice weekly - Absorbent wound dressing changes twice weekly - Post-operative boots If *ulcer heals*, patients assumed to receive no further care If ‘*complicated DFU with infection*’, patients assumed to receive: - Pathology services - Systemic antimicrobials **Optimal care:** Defined according to the National evidence-based guideline on prevention, identification and management of foot complications in diabetes [28] If ‘*uncomplicated DFU*’, patients were assumed to receive: - One-off initial assessment to grade DFU severity by both a podiatrist and GP - Wound debridement weekly - Wound dressing changes consisting of soft-gelling cellulose fibre and polyurethane foam twice weekly - Irremovable pressure offloading device during treatment - Multidisciplinary care from both podiatrist and GP trained in wound management weekly If *ulcer heals*, patients were assumed to receive: - Podiatry consultations every 2 months - One pair of extra-depth footwear per year - Patient education If ‘*complicated DFU with infection*’, patients were assumed to receive: - Pathology services - Topical and systemic antimicrobials - Diagnostic imaging to evaluate suspected osteomyelitis								Consultations with a GP, podiatrist and/or multidisciplinary care team				**Usual care:** *Ongoing costs according to health states (community)* No DFU = $0 Uncomplicated DFU = $302.64 Complicated DFU with infection = $315.83 Post minor amputation = $1,797.50 Post major amputation = $4,934.30 Infected post minor amputation = $315.83 *Initial costs according to health states (community)* Uncomplicated DFU = $67.05 Complicated DFU with infection = $100.80 **Optimal care:** *Ongoing costs according to health states (community)* No DFU = $45.80 Uncomplicated DFU = $504.80 Complicated DFU with infection = $829.59 Post minor amputation = $1,843.30 Post major amputation = $4,934.30 Infected post minor amputation = $829.59 *Initial costs according to health states (community)* Uncomplicated DFU = $296.80 Complicated DFU with infection = $769.90 **Transition costs (hospital):** Minor amputation = $10,640 Major amputation = $23,921 Infected DFU = $16,354 Infected post minor amputation = $25,108			- Australian and international literature - Medicare Benefits Scheme - Pharmaceutical Benefits Scheme - Australian Refined Diagnosis Related Group codes - Expert opinion	
Graves and Zheng (2014) [25]	Direct healthcare costs of chronic wounds in hospital and residential care settings in Australia								Australian hospitals and residential care settings				Based on previous studies, minimum and maximum healthcare costs of DFU in the hospital setting were $5,029 and $32,242, respectively Due to a lack of data, the healthcare costs of DFU in the community setting were used for the residential care setting (i.e. previous studies report costs between $20,343 and $22,310)			- Australian and international literature - Australian Hospital Statistics 2010–2011 - Diabetes Hospitalisations in Australia 2003–2004 - Australian demographic statistics 2011 - Australian residential aged care statistical review 2010–2011	
Zhang et al. (2023) [26]	**Current practice (sub-optimal care):** Defined as not meeting criteria for guideline-based care *DFU episode* One-off costs: Post-op shoe Ongoing costs: Wound management, wound dressing, antibiotics *Healed DFU* One-off costs: $0 (patients wear their own shoes) Ongoing costs: Wound management **Guideline-based care:** *DFU episode* Frequent (≤ 21 days since the previous visit) evidence-based DFU classification documented for 100% of visits in the episode; plus receiving sharp debridement, appropriate wound dressing, antibiotics prescribed (if DFU classified as infected), and knee-high pressure offloading devices during at least 75% of all clinic visits throughout the episode One-off costs: Knee-high removable cast walker offloading device Ongoing costs: Wound management, wound dressing, antibiotics *Healed DFU* Regular (≤ 100 days since the previous visit) evidence-based foot monitoring documented for 100% of visits in the episode; plus receiving sharp debridement, and appropriate footwear during at least 75% of all clinic visits throughout the episode One-off costs: Medical grade extra depth footwear Ongoing costs: Wound management								Diabetic Foot Services				**Current practice (sub-optimal care):** *DFU episode* One-off costs: Post-op shoe ($30) Ongoing costs: Wound management ($186), wound dressing ($1.56), antibiotics ($35.08) x2 *Average outpatient care costs per week:* $176.10 (SD, 185.70) *Healed DFU* One-off costs: None Ongoing costs: Wound management ($186) *Average outpatient care costs per week:* $71.90 (SD, 85.10) **Guideline-based care:** *DFU* One-off costs: Knee-high removable cast walker offloading device ($197) Ongoing costs: Wound management ($186), wound dressing ($11.40), antibiotics ($35.08) × 2 *Average outpatient care costs per week:* $310.50 (SD, 236.70) *Healed DFU* One-off costs: Medical grade extra depth footwear ($176) Ongoing costs: Wound management ($186) *Average outpatient care costs per week:* $124.90 (SD, 112.40) ***Event costs, inpatient (per event):*** Hospitalisation – $15,477 (SD, 14,839) Minor amputation – $30,530 (SD, 14,059) Major amputation – $47,327 (SD, 15,503)			- Australian and international literature - Pharmaceutical Benefits Scheme - Australian Refined Diagnosis Related Group codes - Independent Hospital Pricing Authority - Expert opinion	
**Economic evaluation characteristics**																	
**Author (Year)**	**Study perspective**	**Time horizon**		**Discount rate**			**Reporting of costs**	**Type of model**			**Costs included**				**Measures of health benefit and cost-effectiveness**	**Expected cost savings and health benefits**	**Overall economic evaluation**
Cheng et al. (2017) [22]	Health system perspective	5 years		5%			AUD 2013	Markov model			Consultations with a GP, podiatrist and/or multidisciplinary care team, consumables (e.g. scalpel blades for debridement, wound dressings), pressure offloading devices (e.g. Aircast), footwear, pathology, radiology, antimicrobials, and hospital costs associated with minor or major amputations (e.g. home care, prostheses, inpatient and outpatient care)				QALYs	Overall 5-year cost saving ($9,100 for 35–54 years, $9,392 for 55–74 years and $12,395 for 75 + years) Overall 5-year improved health benefits (0⋅13 QALYs for 35–54 years, 0⋅13 QALYs for 55–74 years and 0⋅16 QALYs for 75 + years)	Cost saving Optimal care dominant in each age group compared to usual care
Graves and Zheng (2014) [25]	Not reported	Not reported		Not reported			USD 2012	Probabilistic model			Hospital separations				Not applicable	Not applicable	Not applicable
Zhang et al. (2023) [26]	Health system perspective	3 years		5%			AUD 2020	Discrete event simulation model			Two categories of costs were considered: (i) average weekly episode care costs (for active DFU or healed DFU) in the outpatient Diabetic Foot Services including healthcare consultations, consumables (such as dressings), pressure offloading devices, footwear and antibiotics and (ii) event costs for hospitalisation (no amputation) and minor / major amputation procedures within the inpatient setting				QALYs ICER NMB	Overall 3-year cost saving of $1,843 and 0.056 QALY per person for 100% guideline-based care, dominating current practice with a NMB of $3,420 Remaining scenarios (40% to 90% guideline-based care) were also dominant relative to current practice with average cost savings between $278 to $1,381 per person (0.011 to 0.045 QALYs)	Cost saving All proportions of guideline-based care (40%-100%) were dominant relative to current practice

*AUD* Australian Dollar, *CKD* Chronic Kidney Disease, *CVD* Cardiovascular Disease, *DFD* Diabetes-Related Foot Disease, *DFU* Diabetes-Related Foot Ulceration, *DM* Diabetes Mellitus, *ESRD* End-Stage Renal Disease, *GP* General Practitioner, *ICER* Incremental Cost-Effectiveness Ratio, *NMB* Net Monetary Benefit, *QALYs* Quality-Adjusted Life Years, *SD* Standard Deviation, *USD* United States Dollar

### Quality appraisal and risk of bias

The Consolidated Health Economic Evaluation Reporting Standards (CHEERS) 2022 checklist [23] was used to appraise study quality and risk of bias. The checklist contains 28 items that are specific to economic evaluations of health interventions [23]. Studies were assessed independently against the CHEERS checklist by two authors (NF and MRK) and a score was calculated out of 28. Based on the methods of a previous systematic review [24], studies were allocated one-point if the criterion was met in full (represented by ✓), 0.5-points if the criterion was partially met (represented by ≠) or 0-points if the criterion was not met (represented by ×) (Table 2). The total score was reduced by one-point for each criterion that was classified as not applicable (represented by N/A). Following the calculation of a percentile score, studies were classified as ‘excellent’ quality if scored 85% or higher, ‘very good’ quality if 70–85%, ‘good’ quality if 55–70% and ‘poor’ quality if below 55% [24].

**Table 2:** Quality assessment using the consolidated health economic evaluation reporting standards (CHEERS) checklist

	**Criterion 1**	**Criterion 2**	**Criterion 3**	**Criterion 4**	**Criterion 5**	**Criterion 6**	**Criterion 7**	**Criterion 8**	**Criterion 9**	**Criterion 10**
	**Title identified as economic evaluation**	**Structured abstract**	**Intro Background and objectives**	**Health economic analysis plan**	**Study population**	**Setting and location**	**Comparators**	**Study perspective**	**Time horizon**	**Discount rate**
Cheng et al (2017) [22]	✓	✓	✓	✓	✓	≠	✓	✓	✓	✓
Graves and Zheng (2014) [25]	✓	✓	✓	✓	≠	✓	N/A	×	×	×
Zhang et al (2023) [26]	≠	✓	✓	✓	✓	✓	✓	✓	✓	✓
	**Criterion 11**	**Criterion 12**	**Criterion 13**	**Criterion 14**	**Criterion 15**	**Criterion 16**	**Criterion 17**	**Criterion 18**	**Criterion 19**	**Criterion 20**
	**Selection of outcomes**	**Measurement of outcomes**	**Valuation of outcomes**	**Measurement and valuation of resources and costs**	**Currency, price date and conversions**	**Rationale and description of model**	**Analytics and assumptions**	**Characterising heterogeneity**	**Characterising distributional effects**	**Characterising uncertainty**
Cheng et al (2017) [22]	✓	✓	✓	✓	✓	✓	✓	×	≠	✓
Graves and Zheng (2014) [25]	N/A	N/A	N/A	✓	✓	✓	≠	×	×	≠
Zhang et al (2023) [26]	✓	✓	✓	N/A	✓	✓	✓	N/A	✓	✓
	**Criterion 21**	**Criterion 22**	**Criterion 23**	**Criterion 24**	**Criterion 25**	**Criterion 26**	**Criterion 27**	**Criterion 28**	**Total score (%)**	**Rating**
	**Approach to engagement with patients and others affected by study**	**Study parameters**	**Summary of main results**	**Effect of uncertainty**	**Effect of engagement with patients and others affected by the study**	**Study findings, limitations, generalisability and current knowledge**	**Source of funding**	**Conflict of interest**		
Cheng et al (2017) [22]	×	✓	✓	✓	×	✓	×	×	22 / 28 (78.6%)	Very good
Graves and Zheng (2014) [25]	×	×	✓	≠	×	✓	×	×	12 / 24 (50.0%)	Poor
Zhang et al (2023) [26]	×	✓	✓	✓	×	✓	✓	✓	23.5 / 26 (90.4%)	Excellent

*Note*. Criterion met in full = 1-point (represented by ✓), criterion partially met = 0.5-points (represented by ≠), criterion not met = 0-points (represented by ×), not applicable = total score reduced by one-point (represented by N/A). Studies classified as ‘excellent’ quality if scored 85% or higher, ‘very good’ quality if 70–85%, ‘good’ quality if 55–70% and ‘poor’ quality if below 55% [24]

### Data synthesis

A descriptive analysis was performed due to limited existing evidence (n = 3) and large heterogeneity between study populations, methodology and data sources to conduct meta-analyses. To ensure that all cost data were reported in this review, the authors of one study [22] were contacted, of which the authors provided their raw data.

## Results

### Study characteristics

**Fig. 1:**
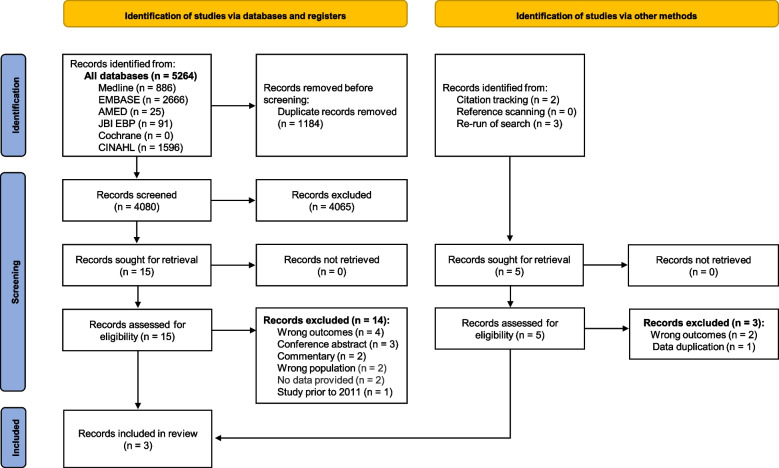
PRISMA flow diagram

The study selection process followed the PRISMA guidelines (Fig. 1). The database searches identified 4,080 unique citations, however only one study initially met the eligibility criteria [22]. Through citation tracking and screening of reference lists, two additional studies were identified [24, 25]. Upon full-text review, one study [25] met the eligibility criteria, while the other [24] was deemed ineligible. After re-running the searches to 20 July 2023, three more articles [9, 26, 27] were identified. One study [26] satisfied the eligibility criteria, while two studies [9, 27] were excluded on full-text review. Overall, only three articles [22, 25, 26] satisfied the eligibility criteria and were included in the review. The characteristics of included studies are presented in Table 1.

Cheng et al. [22] adopted a healthcare system perspective to present a hypothetical cohort of people with diabetes mellitus at high risk of developing DFUs (i.e. those with previous DFU or amputation). Markov cohort simulations were used to evaluate the cost-effectiveness of ‘optimal care’ (including components of foot examination, debridement, wound dressings, pressure offloading, infection management and multidisciplinary care) versus ‘usual care’ for DFD management. The model used seven possible health states including: no DFU, uncomplicated DFU, complicated DFU with infection, post minor amputation, infected post minor amputation, post major amputation, and death. Model inputs were informed by published Australian and international literature, the Medicare Benefits Scheme (MBS), the Pharmaceutical Benefits Scheme (PBS), the Australian Refined Diagnosis Related Group (AR-DRG) codes, and by expert opinion. Separate simulations were also conducted for three age groups (35–54 years, 55–74 years, and 75 + years). The model presented AUD 2013 prices with a discount rate of 5% [22].

The study [22] presented costs for optimal care versus usual care across the three age groups and considered the following DFD costs: consultations with a general practitioner, podiatrist and/or multidisciplinary care team, consumables (e.g. scalpel blades for debridement, wound dressings), pressure offloading devices (e.g. Aircast), footwear, pathology, radiology, anti-microbials, and hospital costs associated with minor or major amputations (e.g. home care, prostheses, inpatient and outpatient care) [22].

Graves and Zheng [25] used a probabilistic model to estimate the direct healthcare costs for treatments of four categories of chronic wounds (i.e. pressure ulcers, DFUs, venous ulcers and arterial ulcers) in hospital and residential care settings in Australia for 2010–2011. The parameters of the model included the incidence of the wounds, and the associated direct healthcare costs in the healthcare setting (e.g. hospital separations). Hospital separation was defined as an episode of admitted patient care, which was either total or part of a hospital stay (e.g. from acute care to rehabilitation). Model inputs were informed from a systematic literature search. Where Australian data were not available, international estimates were used for the economic modelling. Hospital separation data were sourced from the Australian Hospital Statistics 2010–2011 [29]. For patients with diabetes, hospital separation data were derived from Diabetes Hospitalisations in Australia 2003–2004 and Australian demographic statistics 2011 [30, 31]. The hospital separation data for the residential care setting were derived from the Australian residential aged care statistical review 2010–2011 [32]. The model reported the costs in USD 2012 prices. The types of DFU treatments and services provided were not reported [25].

Zhang et al. [26] employed a healthcare system perspective to present a prospective cohort of patients with DFU attending multiple outpatient Diabetic Foot Services. They used a discrete event simulation model based on a state-based Markov model previously described by Cheng et al. [22], to estimate the costs and quality-adjusted life years (QALYs) of seven hypothetical scenarios with increasing proportions of guideline-based care. The scenarios represented discrete episodes of disease which included healed DFU, recurrent DFU, hospitalisation (no amputation), minor amputation, major amputation, and death. The cost-effectiveness of each scenario was estimated by comparing to current practice. The definition of current practice (i.e. 30% of patients receiving guideline-based care and 70% receiving suboptimal care) was based on the observed findings of the authors’ prospective patient cohort [26, 33, 34].

The model incorporated inputs related to time-to-event, resource use and costs, and types of services. Two categories of costs were considered: care costs in the outpatient Diabetic Foot Services and event costs for hospitalisation (no amputation), minor amputation, and major amputation in an inpatient setting. The study used average weekly episode care costs for the outpatient Diabetic Foot Services, which were based on healthcare consultations, consumables (such as dressings), pressure offloading devices, footwear and antibiotics. Event costs associated with hospitalisation and for minor and major amputation procedures were estimated using national hospital pricing data based on AR-DRG codes. The model presented AUD 2020 prices with a discount rate of 5% per year. Comparisons were made between guideline-based care and current practice for the seven scenarios [26].

### Quality appraisal and risk of bias

The included studies [22, 25, 26] were appraised according to the CHEERS 2022 checklist [23]. Table 2 provides the details of the quality appraisal. Cheng et al. [22] scored 22 (78.6%) out of a possible 28 and was rated as ‘very good’ on quality assessment. Graves and Zheng [25] scored 12 (50.0%) out of a possible 24 and was rated as ‘poor’ on quality assessment. Zhang et al. [26] scored 23.5 (90.4%) out of a possible 26 and was rated as ‘excellent’ on quality assessment. Across the three studies, 12 items were met in full (items 2–4, 7,11–16, 23, 26), five items were not met (items 18, 21, 25), and there was variation between studies for the remaining items (Table 2). Overall, items relating to the selection, measurement and valuation of outcomes, the measurement and valuation of resources and costs, the currency, price date and conversions, and the rationale and description of the model were addressed well, while the items concerning heterogeneity and the approach/effect of engagement with patients and stakeholders was lacking.

### Economic evaluation characteristics

The study information, participant characteristics, costs of DFD (including unit costs) and economic evaluation characteristics are summarised in Table 1.

Cheng et al. [22] evaluated the cost-effectiveness of implementing optimal care versus usual care. Overall, the provision of optimal care for DFD using national evidence-based guidelines [28] yielded less costs than providing usual care, where the total five-year cost savings per patient (in AUD 2013) were $9,100 for the 35–54 years age group (0.13 QALYs), $9,392 for 55–74 years (0.13 QALYs) and $12,395 for 75 + years (0.16 QALYs). Five-year cost estimates per patient ranged from $6,681 to $7,066 for optimal care versus $15,781 to $19,461 for usual care. When costs were analysed according to age groups, estimated costs per patient rose with increasing age in both the optimal and usual care groups (Table 3).

**Table 3:** Five-year cost estimates per patient according to age group (AUD 2013 prices)

Age group	Costs (95% CI)	
	**Optimal care**	**Usual care**
35–54	6,681 (2,111 to 15,489)	15,781 (5,514 to 34,707)
55–74	6,943 (2,353 to 16,058)	16,335 (5,962 to 36,096)
75 +	7,066 (2,358 to 16,300)	19,461 (6,604 to 43,385)

Table adapted from Cheng et al. [22]

*Abbreviations*: *AUD* Australian Dollar, *CI* Confidence Interval

*Note*. Costs rounded to the nearest dollar value

Graves and Zheng [25] estimated total hospital care costs (in USD 2012) to be $238.69 million (standard deviation [SD], 123.98 million) and residential care costs to be $11 million (SD, 3.01 million). The total healthcare costs of DFU in both hospital and residential care settings was $249.67 million (SD, 124.02 million) [25].

Zhang et al. [26] used real-world cohort data obtained from the Queensland High Risk Foot Database in Australia. Overall, the provision of 100% guideline-based care (based on international guidelines [35]) over a three-year time horizon yielded a cost saving (in AUD 2020) of $1,843 per patient and an additional 0.056 QALYs per person. The total cost for current practice over a three-year period was estimated to be $49,918 per patient. The breakdown of costs was $15,065 for outpatient DFU care, $27,916 for hospitalisation, $4,521 for minor amputation, and $2,415 for major amputation. Comparatively, the optimal scenario with 100% guideline-based care estimated total costs to be $48,075 per patient; $22,872 attributed to outpatient DFU care, $19,949 for hospitalisation, $3,313 for minor amputation, and $1,940 for major amputation. The cost estimates pertaining to all seven scenarios (i.e. 40%-100% of patients receiving guideline-based care) are outlined in Table 4. In the majority of scenarios, total costs reduced with increasing proportions of guideline-based care (average cost saving between $278 and $1,381 per person). The costs of outpatient DFU care increased with larger proportions of guideline-based care received, but this was offset by reduced costs pertaining to DFU-related hospitalisations as well as for minor and major amputation procedures [26].

**Table 4:** Three-year cost estimates per patient based on proportion of guideline-based care received (AUD 2020 prices)

	**Current practice**	**Percentage of guideline-based care**						
**Scenarios**	**30%**	**40%**	**50%**	**60%**	**70%**	**80%**	**90%**	**100%**
Total costs	49,918	49,639	49,017	48,929	48,853	48,537	48,779	48,075
Outpatient DFU care	15,065	16,210	17,307	18,274	19,372	20,596	21,703	22,872
Hospitalisation	27,916	26,885	25,352	24,319	23,402	22,135	21,533	19,949
Minor amputation	4,521	4,267	4,131	4,093	3,890	3,694	3,481	3,313
Major amputation	2,415	2,277	2,227	2,243	2,189	2,112	2,063	1,940

Table adapted from Zhang et al. [26]

*Abbreviations*: *AUD* Australian Dollar, *DFU* Diabetes-Related Foot Ulceration

## Discussion

### Summary of findings

This systematic review aimed to identify, summarise, and synthesise existing evidence to estimate the costs associated with DFD management in Australia. Our findings revealed a paucity of contemporary evidence on the financial burden of DFD within Australia, particularly in relation to the frequency and variation of services and resources required. Furthermore, there is variation in the reported cost estimates within the Australian literature. Despite the limited evidence at present, this review found that by adhering to evidence-based guidelines, health outcomes can be improved and can bear significant cost savings for the Australian healthcare system.

The variation of reported DFD cost estimates within the Australian literature could be attributed to the differing methodological approaches seen within the included studies, such as the characteristics of study cohorts, the definitions of care/comparators used, the sources of the cost data, and the analyses performed. While the two cost-effectiveness analyses [22, 26] comparing ‘usual care’ to ‘guideline-based care’ used similar modelling methods, the first study [22] used a hypothetical cohort of patients with diabetes at high risk of developing DFUs, while the second study [26] used a large prospective real-world cohort of people with DFU attending Diabetic Foot Services within one state of Australia. As observed in the latter study [26], the method of using individual patient-level data (as opposed to aggregated data) may have better informed the model parameters for guideline-based care versus current practice (i.e. usual care) thus reducing the risk of sampling bias associated with trial-informed time-to-event parameterisation [22, 26]. In addition, the patient-level data allowed for use of a discrete event simulation model in this study, with the flexibility to add specific attributes to each person simulated in the model [26].

When comparing the reported costs for DFD in these cost-effectiveness studies, the first study [22] estimated a total five-year cost per patient (expressed in 2013 AUD) to be between $15,781 and $19,461 (~ $3,156 to $3,892 per year) for usual care, and between $6,681 and $7,066 (~ $1,336 to $1,413 per year) for guideline-based care. The second study [26] estimated a total three-year cost per patient (expressed in 2020 AUD) of $49,918 (~ $16,639 per year) for current practice (i.e. sub-optimal care) and $48,075 (~ $16,025 per year) for guideline-based care. Even despite the inflation of costs between 2013 and 2020, the largely differing cost estimates reported in these studies may also be explained by their respective definitions of ‘guideline-based care’. The first study [22] defined guideline-based care as per the optimal care program outlined in the 2011 National Evidence-based Guideline: Prevention, Identification and Management of Foot Complications in Diabetes [28], while the second study [26] defined guideline-based care as per the core principles of DFU care outlined in international guidelines [35] and included costs associated with healthcare consultations, consumables (i.e. dressings, pressure offloading devices, footwear) and antibiotics. This study [26] further defined healthcare consultations, whereby all visits must have been ≤ 21 days since the previous visit and ≥ 75% of visits must have documented ulcer classification, sharp debridement, appropriate wound dressings, knee-high offloading, and antibiotics prescribed (only if the wound was classified as infected) [26]. Based on the two studies’ definitions of guideline-based care, particularly in relation to the frequency of healthcare consultations, it is apparent that the latter study [26] was more comprehensive in its approach to DFU care than the former study [22]. Hence why the cost estimates may have been significantly larger in this study. It is also important to note that the earlier study [22] reported costs in 2013 AUD, while the other [26] reported costs in 2020 AUD. Therefore, the cost estimates of the earlier study [22] may not be as representative of the current economic burden of DFD. Importantly, both studies [22, 26] demonstrated overall cost savings and improved health outcomes associated with guideline-based care compared to usual care. Accordingly, it is likely that the increase in outpatient costs to perform guideline-based care in these studies were offset by the reduced costs associated with DFU-related hospitalisation and costs of minor and major amputation procedures [26].

The third study [25] included in this review used probabilistic economic modelling to estimate and compare healthcare costs for chronic wounds (including DFUs) in hospital and residential care settings in Australia. In 2012, it was estimated that the total cost for DFU management in hospitals was more than USD$238 million (~ $18,591 per patient), while the total cost in residential care was close to USD$11 million (~ $21,315 per patient). Based on the average conversion rate in 2012 (i.e.1 AUD = 1.0358 USD) [36], this equates to a cost of ~ AUD$17,948 per patient in the hospital setting and ~ AUD$20,578 per patient in the residential care setting. While this study has shown that a large proportion of costs associated with DFD management are incurred in the hospital system (as opposed to residential care services), it is important to consider that these estimates may not be a true reflection of the cost burden in Australia; a large proportion of the inputs for the economic model (i.e. unit costs and incidence of DFU) were informed from international studies, rather than from Australian data. Furthermore, the reported cost estimates showed large standard deviations, which also adds to the uncertainty of the economic modelling in this study [25].

One limitation of all three studies is the reliance on international data to inform specific model inputs, due to the limited published Australian cost and resource utilisation data available. This lack of local data is likely due to fragmented DFD care provision often seen in Australia, but particularly for Aboriginal and/or Torres Strait Islander Peoples [37]. For example, DFD care is commonly shared in the community by general practitioners, podiatrists and nurses, and/or within hospital outpatient specialist clinics and high-risk foot services in the public health system [22, 38]. This lack of local data is further compounded by the limited rebateable services for DFD care, resulting in difficulties for relevant data to be collated and applied to economic evaluations. When comparing Australian and international economic evaluations, it is apparent that data collection methods in Australia may be lacking, particularly when compared to the US and the UK [39, 40]. A more comprehensive system to record item numbers and resources utilised for DFD care within the overall Australian healthcare system may enable a more realistic and representative cost estimate to be determined. With the inception of the National Association of Diabetes Centres (NADC) High Risk Foot Services database [41], and in combination with International Classification of Diseases codes from tertiary care, this could address some of these knowledge gaps, particularly surrounding service provision and resource utilisation within hospital outpatient services across Australia. However, a gap in resource utilisation still remains with DFD services accessed in the community.

Another finding of this review was the variation between the three studies in which Australian healthcare settings (i.e. cost data sources) were included. The first study [22] that used a hypothetical cohort sourced health system data from the Medicare Benefits Scheme (MBS) and the Pharmaceutical Benefits Scheme (PBS), which only partially covers costs of healthcare services and consumables. The second study [26] that used a large prospective real-world cohort presented two categories of care costs, including outpatient DFU services and event costs for hospitalisation and for minor and major amputations in an inpatient setting. This study sourced health system data from the Independent Hospital Pricing Authority and the PBS. For the hospital outpatient data (i.e. from Diabetic Foot Services) this was obtained from only one state in Australia. Therefore, the cost estimates may not be representative of outpatient DFU services throughout Australia. Finally, the third study [25] used total cost data limited to residential care facilities and hospital admissions, therefore, the estimated DFD care costs did not include costs of services and resources from the broader community or outpatient setting. Interestingly, none of the included studies were able to provide an overall cost estimate of DFD with respect to all aspects of care as they did not capture the multi-faceted level of community care throughout the entire patient journey between sectors and over time.

In reference to other Australian studies that were excluded from this review, two out of the three studies (excluded following full-text review) did not provide specific information or costings for DFD management, while the third [24] was a systematic review that included duplicate data from one of our already included studies [22]. Wilkie et al. [27] aimed to determine the actual cost of wound care using a survey to identify the number, type of wounds and their treatment costs including consumables and labour in Australian hospitals, residential aged care facilities, general practitioners, and community providers. Although the data collected on foot ulcers was categorised by the underlying aetiology (e.g. ischaemic, neuropathic, neuro-ischaemic), it was not clear which of these ulcers were directly attributed to DFD, and therefore, this study was excluded. Rather than an economic evaluation of the financial burden of DFD, Ahmed et al. [42] estimated the prevalence of DFD and the sociodemographic and health-related characteristics among people aged 45 years and over in New South Wales, Australia.

There are many challenges when reviewing economic health evaluations due to substantial variability in the standard of care across and within healthcare systems [43]. Economic health evaluations based on assumptions and probabilities of disease states have inherent limitations. They rely on making projections and estimated costs based on various assumptions. These limitations are due to unpredictable factors such as uncertainty of the actual course of the disease, assumptions of human behaviour such as adherence with preventative measures, or data reliability which may affect the accuracy and reliability of the evaluations. It is also acknowledged that systematic reviews of economic evaluations commonly have wide variations in population characteristics, study settings and healthcare systems, therefore reviews are unlikely to generate a one size fits all analysis regarding cost-effectiveness and their comparators [43–45].

### Limitations and strengths

While this systematic review was designed to be comprehensive in capturing contemporary data for the costs associated with DFD management within the Australian context, its findings should be considered in relation to several limitations. First, the quality of the evidence in this review is limited by the small number of included studies. While there were only three economic evaluations identified, two were rated as either ‘very good’ or ‘excellent’ on quality assessment, therefore the findings from these studies [22, 26] are likely to be valid. Second, studies eligible for inclusion in the review were exclusively from Australia, and therefore, cost comparisons with other countries were not extrapolated. Third, searches were limited to the last decade, as we wanted to ensure that cost estimates were representative of present day. Therefore, not all economic evaluations conducted in Australia may have been included in this review. Fourth, as only three studies met the eligibility criteria and there was heterogeneity of the data, pooling of cost data in meta-analyses was not possible. Fifth, we did not find any published Australian data concerning indirect costs (e.g. cessation or reduction of work productivity) associated with DFD management, therefore, only the direct costs have been presented.

That being said, there are several notable strengths of this review. A robust and comprehensive search strategy was employed, and pre-determined decision rules were followed throughout all stages [20]. For example, the study selection, data extraction and quality appraisal steps were conducted by two independent reviewers, with conflicts resolved through consultation with a third party. The reporting of data and the results underwent cross-checking by all authors, ensuring we were transparent and unbiased in our findings.

### Future directions

This systematic review has demonstrated the paucity of evidence regarding not only the costs of DFD management, but also the frequency and variations of services and resources required for management of this patient cohort. There is also a lack of data comparing Australians living in metropolitan, regional or remote communities, and in particular the First Nations population. Of the few studies conducted on DFD prevalence in Australia [8, 11, 42, 46], the data obtained has been predominantly from hospital-based diabetes populations and defined geographic areas, so they may not be reflective of the overall DFD burden within Australian communities [9].

Given the detrimental impact of DFD and its substantial financial burden on the Australian healthcare system, there is a pressing requirement for further economic evaluations utilising up-to-date Australian data. The recent publication of the 2021 evidenced-based Australian guidelines for diabetes-related foot disease [47–52] may inform future Australian economic evaluations concerning the cost-effectiveness of implementing these guidelines (versus usual care), which may prove invaluable for informing national service delivery and improving health and economic outcomes. Within future economic evaluations, it is also crucial to consider the diverse cultural backgrounds, geographic locations and socioeconomic disparities within Australia, as these factors play an important role in evaluating cost implications. Since the Australian DFD guidelines were adapted from international guidelines and tailored to the Australian context by incorporating considerations for First Nations peoples and those living in rural and remote regions, there is now an opportunity to conduct cost-effectiveness analyses for guideline-based care with the Australian context in mind.

To produce robust baseline data across Australia against which improvement to care, access, management and surveillance can be benchmarked, data capture using compatible systems that incorporate the entire patient journey between sectors and over time would be required. Such data could also be used to compare with other countries.

Ideally, the total cost of DFD should be established to understand the true financial impact of DFD to the Australian healthcare system. Data inputs for economic modelling should include the cost of the multidisciplinary team, equipment/consumables, diagnostic tests, medications, hospital and procedure costs, and labour costs. In the absence of alternative data sources to inform these parameters, an additional approach may be to obtain clinical expert opinion to generate estimates of resource utilisation. Multisource data is required to inform real-world resource utilisation and costs associated with care for patients with DFD. This information would enable policy makers the financial incentive to improve access to optimal care for DFD and ultimately reduce the financial burden to both the patient and the Australian healthcare system.

## Conclusions

This review has demonstrated the paucity of contemporary evidence for not only the cost of DFD management within Australia, but also the frequency and variation of services and resources required. While economic evaluations based on assumptions and probabilities of disease states have inherent limitations, they do provide an estimate of the burden, which can be further improved with real-world data. The research to date has highlighted variation in cost estimates for DFU management within Australia. Further research into the economic impact of DFD management and resource utilisation using a national database that captures costs throughout the entire clinical journey between sectors and over time is needed to inform optimisation of national service delivery (e.g. guideline-based care) and to improve health outcomes. Despite the limited evidence at present, this review found that by adhering to evidence-based guidelines, health outcomes can be improved and can bear significant cost savings for the Australian healthcare system.

## Data Availability

All data generated or analysed during this study are included in this published article.

## Abbreviations

AR-DRG: Australian Refined Diagnosis-Related Groups
AUD: Australian Dollar
CHEERS: Consolidated Health Economic Evaluation Reporting Standards
CKD: Chronic Kidney Disease
CVD: Cardiovascular Disease
DFD: Diabetes-Related Foot Disease
DFU: Diabetes-Related Foot Ulceration
DM: Diabetes Mellitus
ESRD: End-Stage Renal Disease
GP: General Practitioner
ICER: Incremental Cost-Effectiveness Ratio
MBS: Medicare Benefits Scheme
NADC: National Association of Diabetes Centres
NMB: Net Monetary Benefit
PBS: Pharmaceutical Benefits Scheme
PRISMA: Preferred Reporting Items for Systematic Review and Meta-Analysis
QALY: Quality-Adjusted Life Years
SD: Standard Deviation
UK: United Kingdom
US: United States
USD: United States Dollar
